# Comparison analysis of short-term outcomes between degradable stent placement and diverting ileostomy in mid-to-low rectal cancer: a retrospective cohort study

**DOI:** 10.1007/s13304-026-02549-2

**Published:** 2026-02-23

**Authors:** Jing Wen, Bo Huang, Minjiang Zheng, Qiushi Huang, Xianzhe Yu, Shan He

**Affiliations:** Department of Gastrointestinal Surgery, West China School of Medicine, Sichuan University, Sichuan University Affiliated Chengdu Second People’s Hospital,Chengdu Second People’s Hospital, Chengdu, China

**Keywords:** Rectal cancer, Degradable stent, Ileostomy, Complications

## Abstract

Anastomotic leakage (AL) is a serious complication of low anterior resection (LAR) of rectal cancer. While ileostomy is the most commonly used method for AL alleviation, it has associated risks of permanent stoma and stoma-related complications, and also requires additional surgery. The aim of this study was to explore the use of a novel technique to protect the anastomosis after LAR. Sixty-five patients were randomly assigned to an LAR combined with degradable stent group (*n* = 32) or an LAR combined with ileostomy group (*n* = 33). The study was conducted between January 2023 and December 2024. Demographic information, laboratory findings, surgical results, and tumor characteristics were recorded. One case of anastomotic stricture (3%) and four cases of intestinal obstruction (12.5%) were observed in the LAR + degradable stent group, and two cases of intestinal obstruction (6%) occurred in the LAR + ileostomy group. No complications, such as abscesses, bleeding, bowel perforation, and anastomotic leakage, were observed in either group. There was no difference in short-term postoperative complications between patients treated with degradable stent or ileostomy, suggesting that the potential of degradable stents as a novel method of preventing anastomotic leakage after low anterior resection of rectal cancer.

## Introduction

 The growing incidence of rectal cancer in the past two decades, together with advances in surigical techniques and the use of preoperative neoadjuvant chemoradiotherapy with total mesorectal excision, has increased the popularity of anus-preserving surgery for rectal cancer [1, 2]. However, the incidence of anastomotic leakage (AL) after laparoscopic anterior resection (LAR) ranges from 3% to 20%, and is associated with increased mortality and reoperation rates, as well as worse long-term prognosis [3, 4]. Various techniques have been proposed to reduce the incidence of AL (e.g., transanal drainage tube placement, diverting stoma) [5, 6]. Although temporary ileostomy is the most commonly used method to prevent serious complications after LAR for rectal cancer, it is associated with various ileostomy-related complications, including peristomal dermatitis, retraction and prolapse, bleeding, hernia, permanent stoma, and additional surgery with stoma [7]. There has long been interest in the development of a new method as an alternative to the traditional ileostomy to protect the colorectal anastomoses.

In this study, we used a biosafety-compatible degradable intestinal stent, containing components that can be developed under X-ray. This was used to block the passage of intestinal contents, together with a mushroom-like tube for diversion of the intestinal contents, thereby avoiding pollution of the anastomosis and thus protecting it. A study by Wu [8] described nine cases in which this degradable stent was applied.

However, more information is required on several aspects: (1) whether the short-term safety of degradable stents is comparable to that of the current gold standard, ileostomy; (2) whether stents reduce stoma-related morbidity and avoid reoperation; and (3) the incidence of stent-specific complications (e.g., intestinal obstruction, incomplete degradation) in a larger cohort.

The primary aim of this study was to compare the short-term safety of LAR combined with degradable stent placement versus the use of LAR together with diverting ileostomy in patients with mid-to-low rectal cancer. The secondary aim was to evaluate differences in operative parameters (e.g., operation time, blood loss), hospital stay, and patient outcomes (avoidance of stoma-reversal surgery). The overall objective was to determine whether the degradable stent could offer a novel therapeutic approach for patients planning to undergo ileostomy.

## Data and methods

### Methods

Eligible patients were randomly assigned to either the LAR + degradable stent group or the LAR + ileostomy group using a computer-generated random number sequence (simple randomization). The randomization was performed by an independent researcher who was not involved in patient recruitment, surgical decision-making, data collection, or outcome assessment to ensure allocation concealment. Group assignments were performed after ensuring that the patients met the inclusion criteria and had completed all preoperative evaluations. Sixty-five patients were enrolled between January 2023 and December 2024, with 32 included in the LAR + degradable stent group and 33 in the LAR + ileostomy group.

This study was approved by the medical ethics committee of Chengdu Second People’s hospital. All patients provided written informed consent for surgery. Tumors were staged according to the rectal cancer guidelines of the National Comprehensive Cancer Network (NCCN) 2023 edition. The diagnosis of rectal cancer was confirmed using preoperative colonoscopic biopsy, ensuring that the tumor was located less than 10 cm from the anus. Preoperative examinations were conducted as a preliminary assessment of whether the conditions were suitable for anal preservation. Chest and abdomen computed tomography (CT) and rectal magnetic resonance imaging (MRI) were performed routinely before surgery. This retrospective cohort study followed the STROBE guidelines for observational studies [9].

### Inclusion criteria

Pathologically confirmed primary single rectal cancer with no distant metastasis; Planned low anterior resection of the tumor; Patient age between 18 and 80 years, with no restriction on sex; A nutritional risk screening score of less than 2; Voluntary participation in the study and the provision of written informed consent for surgery.

**Fig. 1:**
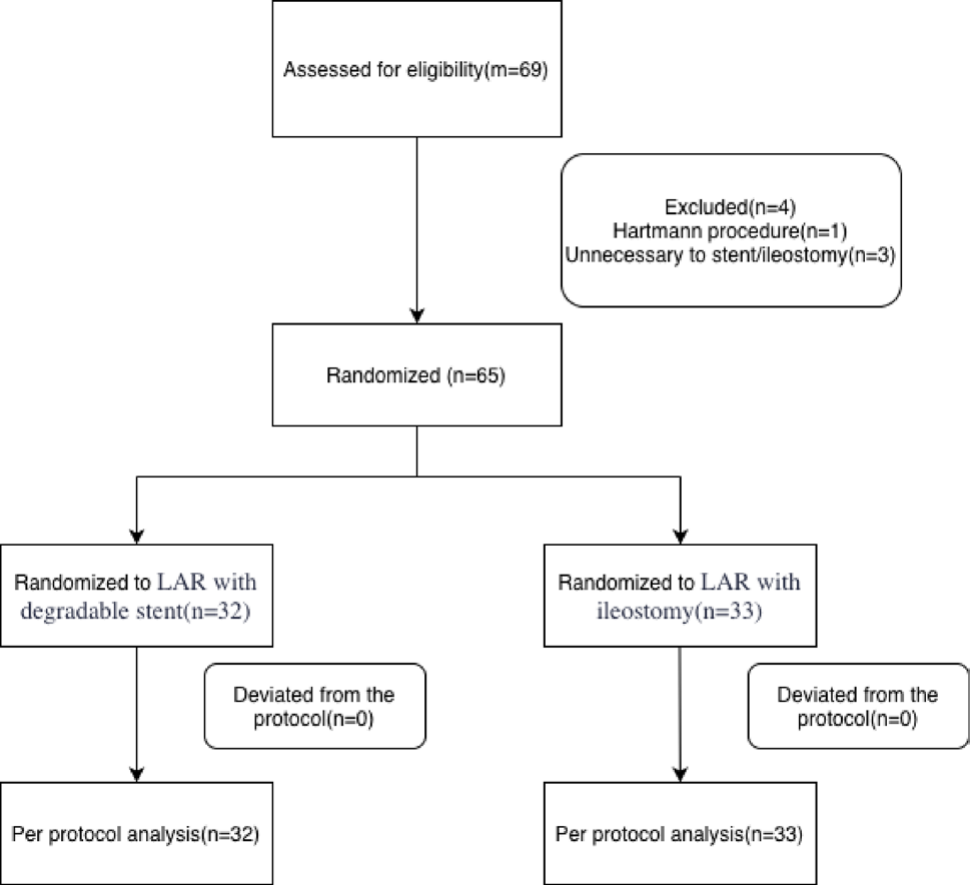
The flowchart of the study

### Exclusion criteria

History of rectal surgery; Familial colorectal polyposis, hereditary non-polyposis colorectal cancer; Active Crohn’s disease or active ulcerative colitis; History of acute cardiovascular or cerebrovascular diseases (such as unstable angina pectoris, acute myocardial infarction) within the previous six months; Continuous use of glucocorticoids within the previous month; Pregnancy, planned pregnancy, or lactation; Severe mental disorders; Severe complications associated with an inability to tolerate surgery or requiring emergency surgery; Intraoperative identification of multiple rectal tumors or distant metastases, or inability to achieve R0 resection; Conversion to laparotomy or other surgical methods resulting in an inability to perform LAR.

### Outcomes

The primary outcome was the incidence of major complications within 30 days of surgery. These complications included anastomotic leakage (AL), abscess, bowel perforation, severe intestinal obstruction (requiring surgical intervention), and bleeding (requiring transfusion or reoperation), assessed according to the Clavien-Dindo classification ≥ IIIa. The secondary outcomes were: (1) Incidence of minor complications (anastomotic stricture, mild intestinal obstruction managed conservatively); (2) operative parameters (total operation time, stent/ileostomy placement time, intraoperative blood loss); (3) length of hospital stay; (4) need for reoperation (including for stoma reversal); and (5) time taken for stent degradation (assessed via imaging).

### Surgical techniques

Step 1: Laparoscopic LAR, involving total mesorectal excision was performed according to the guidelines.

**Fig. 2:**
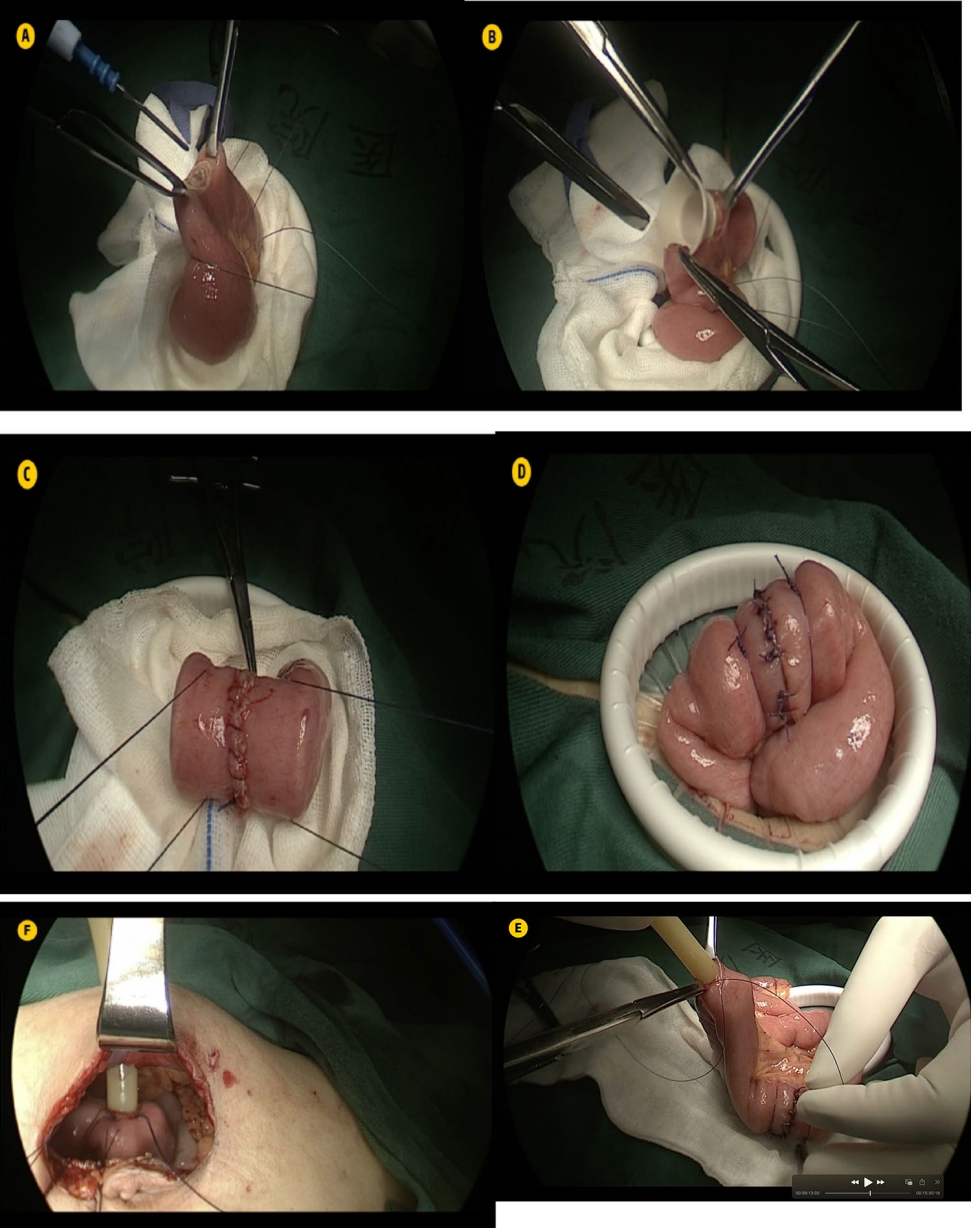
Intraoperative images showing placement of the stent.** A** The ileocecal junction was pulled out through the incision;** B** The degradable stent was placed in position;** C** The intestine was sutured;** D** The stent was held in place using an external tie around the bowel;** E** A mushroom-shaped tube (28 Fr) was placed in the intestine proximal to the stent;** F** This part of the intestine was fixed to the abdominal wall with an absorbable line. The other side of the mushroom-shaped tube was inserted through the right lower abdominal wall, with the image showing the mushroom-shaped tube

Step 2: The surgical steps are illustrated in Fig. 2. The first step, placement of the stent, is a simple procedure that takes 20 min to complete. The ileum is then removed 20 cm from the ileocecal junction, with longitudinal cutting of the intestinal tube to the mesangial margin of the ileum 15 cm from the ileocecal junction, followed by placement of the stent, fixing the intestinal tube on the stent with an absorbable suture, and intermittent suturing of the intestinal tube. Absorbable sutures were used at the 5–10 cm proximal end of the stent to construct a double-loop seromuscular purse-string suture on the mesenteric edge of the intestinal tube. The purse with an inner diameter of 1 cm was prefabricated, and a 28 F intestinal bypass tube was placed in position. The inner and outer loops of the purse-string suture were tightened and knotted in turn, and the shunt tube was led outside the body. Absorbable sutures were used to fix the seromuscular layer of the intestinal tube and the abdominal wall with four stitches placed intermittently along the shunt tube. The shunt tube was fixed to the skin with 2 − 0 non-absorbable suture, and the drainage bag was connected externally. The abdominal incision was closed by layer injection suturing.

**Fig. 3:**
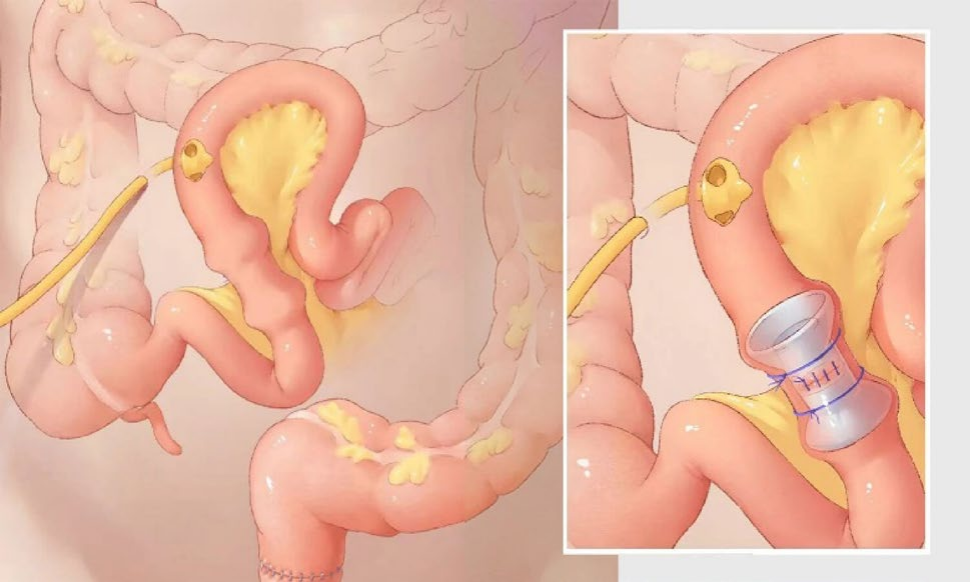
Post-stent placement

Step 3: After surgery, the volume and characteristics of the shunt drainage were monitored and recorded daily, and the shunt was inspected for blockage and displacement. In the case of blockage, saline was used to irrigate the tube. If the blockage cannot be rectified or in the event of complete small-bowel obstruction, surgery should be performed to remove the obstruction and convert to ileostomy. The disintegration of the stent over time was observed by abdominal X-ray or abdominal CT on days 14, 21, and 28 after surgery. If the shape of the stent had become distorted and differed markedly from its original shape, it was considered that the stent had completely disintegrated. Studies have shown that the fragments resulting from stent disintegration can pass smoothly through the ileocecal region without causing damage to the intestine or anastomosis [10]. Gastroenterography was performed through the bypass tube. If no anastomotic leakage was observed, the bypass tube was clamped and removed 3 days later. Postoperative follow-up was performed regularly every three months.

### Statistical analysis

Statistical analysis was performed using SPSS 19.0 (IBM Corp., Armonk, NY, USA). Categorical variables are presented as numbers (%), while continuous variables are expressed as mean (standard deviation) or median [interquartile range], depending on the data distribution. Categorical data were compared using chi-square tests, while normally distributed continuous data were compared using t-tests and non-normally distributed data using Mann-Whitney U tests. *P*-values < 0.05 were considered statistically significant.

## Result

### Patient characteristics

From January 2023 to December 2024, 69 consecutive patients were enrolled in the study, while 4 patients were excluded for various reasons (Fig. 1). The 65 patients.

were randomly assigned to either the LAR + degradable stent group (*n* = 32) or the LAR + ileostomy group (*n* = 33). The two groups were comparable in terms of patient characteristics and the distance of the tumor from the anus (Table 1).

**Table 1:** Patient characteristics

Characteristics	Degradable stent		Ileostomy	*p*-value
Age, years		67.19 ± 11.86	67.24 ± 11.33	0.985
Gender	male	13(40.6%)	25(75.6%)	0.159
	female	19(59.4%)	8(24.4%)	
BMI, kg/m^2^		23.60 ± 3.16	24.18 ± 3.36	0.479
Albumin, g/L		41.78 ± 4.46	39.76 ± 3.76	0.052
Hemoglobin, g/L		126.84 ± 23.14	117.36 ± 30.68	0.165
Distance of tumor to anus, cm		5.16 ± 1.11	5.67 ± 1.36	0.103

Age, Gender, BMI, Albumin, Hemoglobin.* P* values are derived from T- tests. Distance of tumor to anus.* P* values are derived from U- tests. *Values are statistical significance (*p*<0.05)

### Operative parameters

Table 2 summarizes the pathological characteristics of the patients as well as the operative parameters. There was no significant difference in TNM staging between the two groups. In the LAR + degradable stent group, 4 patients (12.5%) received preoperative chemotherapy, while in the LAR + ileostomy group, 5 patients (15.1%) underwent preoperative chemotherapy. Significant differences were found between the LAR + degradable stent and LAR + ileostomy groups in terms of total operation time (240.47 ± 60.14 vs. 298.39 ± 61.12, respectively) and stent placement time (29.56 ± 7.02 vs. 45.76 ± 28.51, respectively), while there were no significant differences in blood loss or hospital stay.

**Table 2:** pathological characteristics and operative parameters

characteristics	Degradable stent		Ileostomy	*p*-value
*Preoperative neoadjuvant*				0.32
Absent	28(87.5%)		28(84.8%)	
Chemotherapy	4(12.5%)		5(15.2%)	
*Radio-chemotherapy*	0		0	
*T staging*				0.528
0	0		0	
1	0		2(6%)	
2	8(25%)		5(15.2%)	
3	19(59.4%)		18(54.6%)	
4	5(15.6%)		8(24.2%)	
*N staging*				0.079
0	8(25%)		15(45.5%)	
1	17(53.1%)		14(42.4%)	
2	7(21.9%)		4(12.1%)	
*M staging*				
0	32(100%)		33(100%)	
1	0		0	
*TNM staging*				0.077
0	0		0	
I	0		5(15.2%)	
II	8(25%)		9(27.2%)	
III	24(75%)		19(57.6%)	
IV	0		0	
Operating time, min	240.47 ± 60.14		298.39 ± 61.12	*0.001
stent placement time/ileostomy time	29.56 ± 7.02		44.82 ± 8.34	*0.001
Blood loss, mL	32.03 ± 20.23		45.76 ± 28.51	0.029
Hospital stay, day	22.72 ± 5.51		21.09 ± 4.24	0.186

Hospital stay.* P* values are derived from T- tests. Preoperative neoadjuvant, T staging,N staging, M staging, TNM staging, Operating time,Stent placement time/ileostomy time, Blood loss, .* P* values are derived from U- tests. *Values are statistical significance (*p*<0.05)

### 30-Day morbidity

In terms of postoperative complications (Table 3), the stent group showed two types of short-term complications. Specifically, there was one case of anastomotic stricture (3%) and four cases of intestinal obstruction (12.5%). All four cases of intestinal obstruction involved mild-to-moderate partial intestinal obstruction, which was most likely caused by obstructed drainage of the intestinal juice through the mushroom-like tube. The issue was solved by irrigation of the mushroom-like tube with normal saline. At the one-month postoperative follow-up, one patient was found to have an anastomotic stricture, which was relieved by transanal dilation. A subsequent gastroenterography indicated an absence of anastomotic leakage and anastomotic stricture. Therefore, we recommend that weekly rectal examinations during the postoperative period could effectively prevent the development of anastomotic stricture. The ileostomy group had two cases of intestinal obstruction (6%), with no anastomotic stricture or other complications. These two cases were managed conservatively or with minimally invasive interventions, with no need for reoperation. No other problems were seen in either of the groups (e.g., anastomotic leakage, abscess, bleeding, bowel perforation).

**Table 3:** 30-day morbidity

complications	LAR + stent		LAR + illeotomy		*p*-value
	*n* = 32	%	*n* = 33	%	
Anastomotic leakage	0	0	0	0	
Intestinal obstruction	4	12.5	2	6	0.306
Anastomotic stricture	1	3.1	0	0	0.37
Abecess	0	0	0	0	
Bleeding	0	0	0	0	
Bowel perforation	0	0	0	0	

Anastomotic leakage, Intestinal obstruction, Anastomotic stricture, Aecess,Bleeding, Bowel perforation.* P* values are derived from Chi-square tests

## Discussion

Several methods have been explored to minimize the risk of AL in patients following LAR; these mainly involve the diversion of feces or reductions in intestinal pressure [10, 11, 12]. Yeow et al. conducted a systematic review and network meta-analysis, comparing different strategies for the prevention of AL in patients following LAR, and found that stoma diversion was effective in preventing both AL and reoperation. However, the disadvantage of this method is that it may result in a permanent stoma, stoma-related complications, and the risk of further surgery to accept the reverse stoma [13]. In addition, while ileostomy after rectal cancer surgery cannot reduce the incidence of AL and related morbidity, it is associated with a higher incidence of stoma-related morbidity and a one-year stoma rate [14]. The common disadvantages of placing an anal canal include the development of perianal abrasions and early dislocation [6].

Therefore, we utilized a degradable stent to divert the feces, hoping that this would have the same effect as ileostomy, while also reducing complications associated with a diversion stoma [15]. The literature shows that degradable stents have acceptable biosafety [16]. The barium sulfate used in this study enabled X-ray monitoring of stent degradation, showing that the stent was effective in preventing entry of the intestinal content into the distal intestine, thereby achieving the effect of intestinal content bypass. In general, the use of collapsible stents is feasible in clinical applications.

Preoperative neoadjuvant chemoradiotherapy has been shown to increase the incidence of anastomotic leakage [17]. In this study, 32 patients underwent LAR with stent, of whom four underwent preoperative chemotherapy. No anastomotic leakage was observed in these patients. Although fecal diversion cannot prevent anastomotic leakage, it can help reduce the severity of inflammation should leakage occur. Based on the degradable stent, we used mushroom-like tubes for diversion of the intestinal contents. As the stent is composed of biodegradable material, it can decompose into tiny fragments under the action of intestinal peristalsis in the third or fourth week after surgery. A cohort study by Tong et al. [18] indicated that compared with ileostomy, the stent-based diversion technique did not increase complications due to is effectiveness in reducing stoma-related complications. This method avoids re-hospitalization for ostomy and complications resulting from ileostomy, thereby reducing medical costs and improving the patient’s quality of life. Although four patients experienced abdominal distension due to obstructed drainage of the intestinal fluid through the mushroom-like tube, the symptoms were relieved after saline irrigation. It is recommended that patients should eat a low-fiber diet until the stent is completely degraded. Compared with patients with ileostomy, patients with degradable stent placement showed no increased incidence of postoperative intestinal obstruction.

**Fig. 4:**
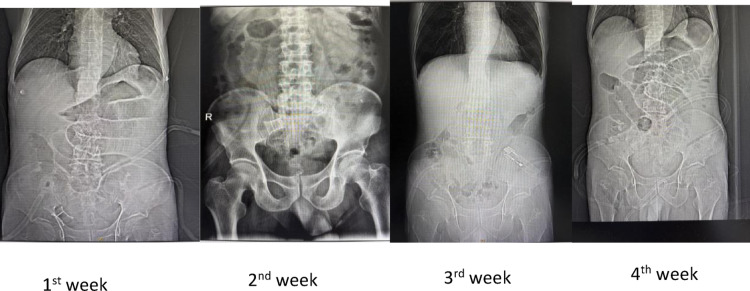
X-ray monitoring of stent degradation. The stents of the patient were degraded completely within 4 weeks

The procedure involved in stent placement is simple. This study found that the operation time required for stent placement was significantly shorter than that of ileostomy, while there was also a significant reduction in the amount of bleeding, indicating that stent placement is a simple and generalizable method. Compared with the placement of an anal canal, the degradable stent does not cause anal pain, shedding of the anal canal, or other risks.

The study has inherent limitations. These include its single-center retrospective design, small sample size, short-term follow-up, and lack of subgroup analyses for high-risk populations. We intend to address these limitations in future studies by conducting a multicenter prospective randomized controlled trial with a larger sample size, extended follow-up (2–5 years), and analyses of predefined subgroups to identify high-risk populations. This will validate the long-term safety, efficacy, and generalizability of degradable stents as an alternative to ileostomy to protect the anatomosis in surgery for mid-to-low rectal cancer.

## Conclusion

Degradable stent placement represents a safe, technically feasible alternative to ileostomy for preventing AL in surgery for mid-to-low rectal cancer, showing reduced operative time and stoma-related morbidity. Long-term functional outcomes require further investigation. Although this exploratory study indicated the effectiveness of degradable stents as a novel protective procedure in patients with LAR, assisting in reducing the severity of AL, more research is needed to determine whether the procedure is feasible in other populations of patients with rectal cancer.

## Data Availability

The datasets used and/or analyzed during the current study are available from the corresponding author on reasonable request.

## Abbreviations

AL: Anastomotic leakage
LAR: Low anterior resection
TME: Total mesorectal excision Acknowledgments
